# Nurses' Registration

**Published:** 1919-06-28

**Authors:** Donald MacMaster

**Affiliations:** (Chairman Standing Committee E.) Merlewood, Virginia Water, June 11.


### Nurses' Registration.

The following letter continues the correspondence in the *Times* which we published in THE HOSPITAL of June 7 and 14, pp. 251 and 275:—

SIR,—I have just read Sir Henry Burdett's letter in your issue of the 9th inst. Any report that I suggested that the Central Committee's Bill should be referred back to the Committee, on the ground that "the Committee had been misled," lacks foundation. In view of the numerous amendments put upon the paper, there may be ground for further consideration in Committee or in the House—but that is another and a different matter.

Yours sincerely,

DONALD MACMASTER.

(Chairman Standing Committee E.)

Merlewood, Virginia Water, June 11.

(*Times*, June 12.)

